# A method for checking high dose rate treatment times for vaginal applicators

**DOI:** 10.1120/jacmp.v2i4.2595

**Published:** 2001-09-01

**Authors:** Charles S. Mayo, Kenneth Ulin

**Affiliations:** ^1^ Department of Radiation Oncology UMASS Memorial Healthcare Worcester Massachusetts; ^2^ Department of Radiation Oncology New England Medical Center Boston Massachusetts

**Keywords:** HDR, brachytherapy, vaginal cylinder, TG‐59

## Abstract

A method is presented for checking the treatment time calculation for high dose rate (HDR) vaginal cylinder treatments. The method represents an independent check of the HDR planning system and can take into account nonuniform isodose line coverage around the cylinder. Only the air kerma strength of the source and information that is available from the written directive are required. The maximum discrepancy for a representative set of cylinder plans done on a Nucletron unit was 5%. A working HTML JavaScript program is included in the Appendix.

PACS number(s): 87.53.–j, 87.90.+y

## INTRODUCTION

The report of AAMP task group TG‐59^1^ includes a recommendation to check high dose rate (HDR) treatment time calculations provided by the treatment planning system. Ideally the calculation should be independent of the planning system. One approach is to calculate and sum the dose contribution from each dwell position to a calculation point. For this method the coordinates and dwell times of each source position must be determined. A typical vaginal cylinder application has 10–20 active dwell positions. The time required to carry out such second checks in a busy clinic renders this method impractical.

A recent report describes a computerized method for doing a check of HDR calculations using the treatment unit data file as the primary input to their program.^2^ Various hand calculation methods for checking HDR calculations have also been described.^3–^, ^6^ Saw *et al.* described a method for checking HDR treatment time calculations using an LDR planning system.^7^ Published hand calculation methods for checking single catheter treatments generally assume that dose is prescribed to a uniform depth around the applicator. In our experience, however, vaginal cylinder prescriptions often specify that the prescribed dose be delivered to a depth of 0.5 cm near the vaginal apex (the proximal end of the cylinder) and to the surface at the distal end of the cylinder. We present an empirical method for calculating the total dwell time for either uniform or nonuniform coverage around the cylinder. The only information required is the air kerma strength of the source, the prescribed dose, the active length of the cylinder, and the specification of where the dose is prescribed. With the exception of the air kerma strength all of the required information is present on the written directive.

## METHODS AND MATERIALS

The method described here is based on the use of a treatment time factor (*K*). The treatment time factor is defined in terms of total treatment dwell time (*TT*), prescribed dose (*D*), and the air kerma strength (AKS) of the HDR source as

(1) K=TT×AKS/D.

The reader will recognize this expression as analogous to the classical mg/hr per 1000 rad formalism. If Eq. (1) is rewritten as

(2) TT=K×D/AKS,

it is clear that estimating the total treatment dwell time with acceptable accuracy requires an accurate means of determining *K*.

**Figure 1:**
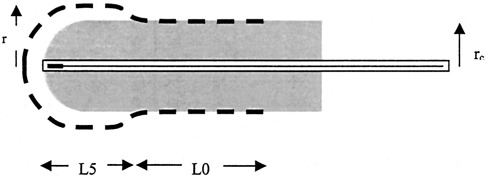
The prescription isodose line (dashed) is illustrated with respect to the HDR cylinder surface. The first dwell position of the source is located at the end of the cylinder. In this illustration the dose has been prescribed to a depth of 5 mm from the surface for a segment equal to L5 and to the surface for the segment L0.

Figure 1 illustrates a prescription to a depth of 0.5 cm at the vaginal apex diminishing to 0 cm at the distal end of the cylinder. The active lengths prescribed to 0.5‐cm depth and to the surface are represented by $L_{5}$ and $L_{0}$, respectively. The prescription radius and the cylinder radius are *r* and $r_{C}$, respectively.

The treatment time factor (*K*) is modeled as the sum of three components:

(3) $$K=a(r,r_{C})+b(r)L_{5}+b(r_{C})[L_{0}-l(r_{C})].$$

The first term of this equation calculates the contribution from the hemispherical end of the cylinder. The second term represents the contribution from the portion of the active length prescribed to a depth of 5 mm (i.e., $r=r_{C}+5$). The third term is the contribution from the segment prescribed to the surface of the cylinder ($r=r_{C}$). The length of this segment ($L_{0}$) is diminished by $l(r_{C})$ to account for the overlap contribution of the $L_{5}$ segment.

The first term is described by a quadratic equation, evaluated as a function of both the cylinder radius and the prescription radius at the hemispherical end of the cylinder:

(4) $$a(r,r_{C})=\alpha_{2}r^{2}+\alpha_{1}r-\alpha_{0}(r_{C}).$$

The proportionality constants for the $L_{0}$ and $L_{5}$ segments are determined from an equation which is linear in the prescription radius

(5) $$b(r)=\beta_{1}r-\beta_{0}.$$

The adjustment factor applied to $L_{0}$ is represented by a quadratic function of the cylinder radius:

(6) $$l(r_{C})=\lambda_{2}r_{C}^{2}-\lambda_{1}r_{C}+\lambda_{0}.$$

**Figure 2:**
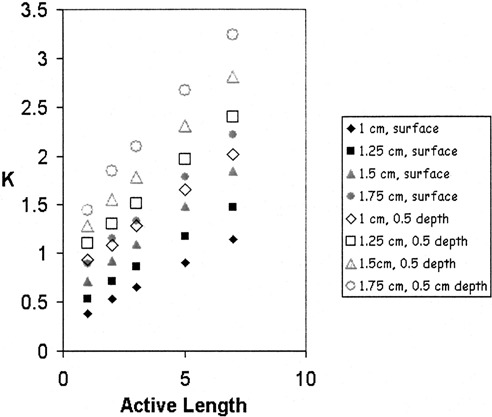
(Color) Treatment time factors, K, determined from the HDR treatment planning system are plotted with respect to active length for doses prescribed to the surface (solid symbols) and to a depth of 5 mm (empty symbols). K factors were calculated for cylinder radii of 1 (diamond), 1.25 (square), 1.5 (triangle), and 1.75 (circle) cm.

Values of the coefficients in these equations were determined from fits to treatment time factors calculated for a clinically relevant range of *r*, $r_{C}$, $L_{0}$, and $L_{5}$ values. Figure 2 illustrates the data set used for doses prescribed to the surface and to a depth of 0.5 cm for the range of available cylinder diameters and active lengths from 1 to 7 cm. Calculations were carried out with the HDR treatment planning system and verified by hand calculations.

The data in Fig. 2 were fit to a linear equation as a function of active length. The intercept coefficients were then grouped by cylinder radius and fitted to a quadratic equation with respect to the treatment radius to determine the coefficients in Eq. (4). The slope coefficients were fitted to an equation linear in the treatment radius to determine the values of coefficients in Eq. (5).

To determine the coefficients for the length adjustment factor, $l(r_{C})$, total treatment factors were calculated for total active lengths of 3 and 5 cm. The length segment prescribed to 0.5 cm depth was equal to 2 cm for the 3 cm active length and 3 cm for the 5 cm active length. The three data points for each cylinder radius were then fitted to Eq. (3) to find a value of l for each cylinder radius. These values of *l* were then fitted to a quadratic equation in $r_{C}$ to determine the coefficients in Eq. (6).

The accuracy of the Eqs. (2)–(6) for predicting the total treatment time was examined in two ways. First, total treatment times were calculated for the sample data set used to determine the coefficients in Eqs. (4)–(6). These were compared to the total treatment times calculated with the treatment planning system. Second agreement of calculations with actual treatment times for a random sample of 15 patients was examined. In this sample, only patients whose prescriptions included a mix of depths, as in Fig. 1 were included.

## RESULTS

In determining the coefficients for Eq. (4), only the $\alpha_{0}$ coefficient demonstrated significant dependence on prescription depth. Therefore, average values were used for the $\alpha_{1}$ and $\alpha_{2}$ coefficients. For the Eq. (5) coefficients, no significant dependence on treatment depth was observed. Values for all coefficients are provided in Table I.

**Table I:** Coefficients determined from fits to data in Fig. 2, enable calculation of total treatment times for prescriptions typically encountered in our clinic.

$\alpha_{0}=$	−0.1271	for $r_{C}=r$
$\alpha_{0}=$	0.0669	for $r_{C}=r-0.5\;\text{cm}$
$\alpha_{1}=$	0.3128
$\alpha_{2}=$	0.0865
$\beta_{0}=$	−0.0117
$\beta_{1}=$	0.1327
$\lambda_{0}=$	4.2686
$\lambda_{1}=$	−2.5726
$\lambda_{2}=$	0.3429

The tests of the accuracy of the calculation method were favorable. Agreement with total treatment times in the data set used for fitting was better than 4%. In the comparison with data set of patient treatment times, the average discrepancy in this test was 1.5%, and the maximum discrepancy was 5.1%. Since the dose gradient at the surface of the cylinder is on the order of 10% per millimeter, this indicates excellent agreement with the prescription depths.

## DISCUSSION

While a calculation method may provide an accurate calculation check of total treatment time computed with an HDR planning system, it is only clinically useful if it can be implemented in a way that makes it accessible and fast. We accomplished this with a web page based computer program. A fully functional example that uses a JavaScript program is provided in the Appendix. To use it, save the file as a text file with the name “HDRCALC.HTML.” The program may then be run from within Windows by double clicking on it. The web browser will be invoked automatically to open and run the program.

By using a JavaScript embedded in an HTML file to encode a numerical model for calculating total treatment times, we achieved two improvements over an alternative approach such as creating a look up table for total treatment times. The method described requires a relatively small number of treatment time calculations. Best fit parameters were derived from 52 treatment time calculations. If three rather than five active lengths had been utilized in Fig. 2, similar agreement might have been obtained with 24 data sets. In contrast, substantially more calculations would be required for a look up table approach. For example, using a 3‐cm diameter cylinder with a total active length of 5 cm, the total treatment time increases with the active length by ~1.5% mm. Based on this observation, the reader may show that several hundred treatment time calculations would therefore be needed to achieve 3% accuracy in a look up table that accommodated the four cylinder sizes examined, total active lengths of 1–7 cm, and compound prescription depths of 0 to 0.5 cm.

The second improvement was adopting a computer method that is platform independent, free of charge, suited to use on a department's intranet and readily utilized even by physicists with limited programming skills. HTML files with embedded JavaScripts run in web browsers on Windows and Linux based PCs, Macintosh computers, and on pocket PCs. The more conventional approach of creating an application on a spread sheet requires that each user have a copy of the base application (EXCEL, LOTUS 1‐2‐3, Quattro, etc.) installed on their hard drive or accessible from their network. As a result, that approach is more expensive and tends to limit users to a single platform and operating system. Since the JavaScripts are part of HTML files, they do not require any more than a text editor and web browser to create and run. The coding syntax of JavaScript is similar to that of C+ +. For users more accustomed to BASIC programming, VBScripting is an alternative.

## CONCLUSIONS

A method has been described for estimating the total treatment time for vaginal cylinder HDR treatments. The method can account for a prescription that specifies either uniform or nonuniform isodose coverage around the cylinder. By shifting the focus of calculation checks from point dose calculations based on the treatment array of dwell times and positions to the total treatment time, we have been able to implement an expeditious method of checking HDR cylinder calculations. It has the added advantage of enabling staff to accurately predict treatment times prior to the actual plan.

Coefficients were obtained using treatment times calculated for both for the Nucletron V2 and Classic HDR brachytherapy systems. Users of other systems should carry out similar calculations to determine the best fit parameters for their systems.

## Appendix

<!DOCTYPE HTML PUBLIC “‐//W3C//DTD HTML 4.0 Transitional//EN”>

<html>

<head></head>

<body bgcolor= “#ffe7a6”>

<h2>HDR Cylinder Treatment Time Check(</h2>

<script language= “JavaScript”>>

        function calctime(){

            var strength=eval(document.calcform.strength.value)

            if (document.calcform.units[1].checked){

            strength= strength*0.4208

            }

            var dose=eval(document.calcform.dose.value)

            var *r*_*C*_=eval(document.calcform.dc.value)/2.0

            var al=eval(document.calcform.al.value)

            var l5=eval(document.calcform.l5.value)

            r=rc + 0.5

            Bf=0.1327*(rc+0.5)–0.0117

            Bz=0.1327*rc–0.0117

            A=0.0865*r*r+0.3128*r+((l5<=0)?–0.1271:0.0669)

            l0=al–l5

            l=0.3429*rc*rc‐2.5726*rc+4.2686

            l=((r>=rc)?l:0)

            l0=l0–l

            var k=A+Bf*l5+Bz*10

            document.calcform.tt.value=Math.ceil(10*k*dose/strength)/10

            document.calcform.plantt.value = “”

            document.calcform.pdiff.value= “”

         }

         function compare(){

            var tt=document.calcform.tt.value

            var plantt=document.calcform.plantt.value

            if((tt>0)&&(plantt>0)){

            document.calcform.pdiff.value=Math.ceil(1000*(plantt‐tt)/plantt)/10

            }

        }

        function cleardata(){

            document.calcform.tt.value= “”

            document.calcform.plantt.value = “”

            document.calcform.pdiff.value= “”

        }

</script>>

<form name = “calcform”>

<table>

<tr>

     <td>Source strength</td>

     <td><inputtype=“text” name=“strength” size=“4” onfocus=“cleardata()”>

        <inputtype=“radio” name=“units” onfocus=“cleardata()” checked

        >cGy*m<sup>2<</<sup>/hr<input type= “radio” name= “units” onfocus=

           “cleardata()”>Ci</td>

</tr>

<tr>

     <td>Input the prescribed dose (cGy)</td>

     <td><inputtype=“text” name=“dose” size=“4” onfocus=”cleardata()”></td>

</tr>

<tr>

     <td>Input the cylinder diameter (cm)</td>

     <td><inputtype=“text” name=“dc” size=“4” onfocus=“cleardata()”></td>

</tr>

<tr>

     <td>Input the active length (cm)</td>

     <td><inputtype=“text” name=“al” size=“4” onfocus=“cleardata()”></td>

</tr>

<tr>

     <td>Input the length of the 0.5 cm depth segment (cm)</td>

     <td><inputtype=“text” name=“l5” size=“4” onfocus=“cleardata()”></td>

</tr>

<tr>

     <td><inputtype = “button” name = “calcbutton”

            value=“Calculate the Total Treatment Time” OnClick=calctime()></td>

</tr>

<tr>

     <td>Total Treatment Time (sec)</td>

     <td><inputtype = “text” name=“tt” size=“4”></td>

</tr>

<tr>

     <td>Total Treatment Time calculated by the planning system (sec)</td>

     <td><inputtype = “text” name= “plantt” size=4)></td>

</tr>

<tr>

     <td><input type=“button” name=” calcdiff” value=” Calculate percent difference”

            onclick= “compare()”</td>

</tr>

<tr>

     <td>Percent difference between times</td>

     <td><inputtype = “text” size=4 name = “pdiff”></td>

</tr>

</table></form>

</body>

</html>
